# Resting state functional connectivity data supports detection of cognition in the rodent brain

**DOI:** 10.1016/j.dib.2016.03.041

**Published:** 2016-03-15

**Authors:** Fatima A. Nasrallah, Xuan Vinh To, Der-Yow Chen, Aryeh Routtenberg, Kai-Hsiang Chuang

**Affiliations:** a MRI Group, Singapore Bioimaging Consortium, A^⁎^STAR, Singapore; b Psychology, National Cheng-Kung University, Tainan, Taiwan; c Psychology, Neurobiology and Physiology, Northwestern University, Evanston, IL, USA; d Clinical Imaging Research Centre, National University of Singapore, Singapore; e Department of Physiology, Yong Loo Lin School of Medicine, National University of Singapore, Singapore; f Physiology, Feinberg School of Medicine Northwestern University, Chicago, IL, USA; g The Queensland Brain Institute, the University of Queensland, Queensland, Australia

**Keywords:** MRI, Morris water maze, Functional connectivity, Resting state, Learning and memory, Neural plasticity

## Abstract

Learning is a process which induces plastic changes in the synapses and connections across different regions of the brain. It is hypothesized that these new connections can be tracked with resting state functional connectivity MRI. While most of the evidence of learning-induced plasticity arises from previous human data, data from sedated rats that had undergone training for either 1 day or 5 days in a Morris Watermaze is presented. Seed points were taken from the somatosensory and visual cortices, and the hippocampal CA3 to detect connectivity changes. The data demonstrates that 5-day trained rats showed increased correlations between the hippocampal CA3 and thalamus, septum and cingulate cortex, compared to swim control or naïve animals. Seven days after the training, persistent but reorganized networks toward the cortex were observed. Data from the 1-day trained rats, on the contrary, showed connectivity similar to the swim control and less persistent. The connectivity in several regions was highly correlated with the behavioral performance in these animals. The data demonstrates that longitudinal changes following learning-induced plasticity can be detected and tracked with resting state connectivity.

**Specifications Table**

**Table:** 

Subject area	*Neuroscience*
More specific subject area	*Functional connectivity in learning*
Type of data	*Figures*
How data was acquired	*Resting state functional MRI*
Data format	*Matlab, Analyzed data*
Experimental factors	*Connectivity networks and Watermaze training*
Experimental features	*Resting state fmri can be used to perform connectomics on data*
Data source location	*Singapore Bioimaging Consortium, Singapore*
Data accessibility	*Data is within this article.*

**Value of the data:**

• The datasets provided would serve as a basis platform to compare with other cohorts of disease. The data sets that we have provided may form as control data sets for those investigating resting state networks in disease cohorts.
• Additionally, the data can serve as a baseline from which others can build on to investigate brain connectivity networks in learning and memory and investigate memory consolidation.
• Lastly, these data can be used to identify possible subgroups for further analysis or collaboration: the given sample sizes can be used as important inputs for power calculations needed to determine the feasibility of a substudy of this cohort.

## 1 Data

The data in this report represent a detailed characterization of the brain networks in a group of normal Wistar rats after being trained on a spatial Morris Watermaze task. The data represents resting state connectivity correlation coefficients between multiple selected regions of interest extracted from animals that have either been trained for 1 day or for 5 days at both 1 days after training or 7 days after training.

## 2 Experimental design, materials and methods

### 2.1 Experimental design

The study was comprised of 2 phases; in phase 1, rats were trained on a Morris Watermaze (MWM) [1] for a period of 5 days or 1 day. In phase 2, the same rats were scanned using Magnetic Resonance Imaging (MRI) at 1 day and 7 days after the last day of MWM training. In total, 45 male adult Wistar rats (350–400 g) were included in the study and were subdivided into five groups: naive control (*n*=10), 5-day trained (*n*=9), 5-day swim control (*n*=8), 1-day trained (*n*=9) and 1-day swim control (*n*=9). All experiments were compliant with the National Advisory Committee for Laboratory Animal Research guidelines and approved by the Institutional Care and Use Committee (Biomedical Sciences Institutes, Singapore).

### 2.2 Probe tests for evaluation of medetomidine effect on memory

Medetomidine, an α2 adrenergic agonist, was used as sedative during the rsMRI experiments. Because alpha2 agonists have been reported to impair learning and memory, probe tests were conducted in 3 groups of rats after 5 days of MWM training to evaluate the effect of medetomidine on memory: Group A. Rats were injected with 0.1 mg/kg/h medetomidine (*n*=5) for 1 h or saline *(n*=5) on day 1 after the training. Probe test was conducted in the watermaze without the platform on day 2. Group B. Rats were injected with 0.1 mg/kg/h medetomidine (*n*=5) for 1 h or saline (*n*=5) on day 7 after the training. Probe test was conducted in the watermaze without the platform on day 8. Group C. Rats were injected with 0.1 mg/kg/h medetomidine (*n*=5) for 1 h or saline (*n*=5) on both day 1 and day 7 after the training. Probe test was conducted in the watermaze without the platform on day 8. (refer to Fig. 2 for results).

## 3 Materials and methods

### 3.1 Behavior

The trained rats underwent a hidden platform task for 1 day or 5 consecutive days, with the swim control rats matching the swimming time of the trained rats in the MWM without the platform. The rats were habituated to the pool one day before training. During the training, rats were randomly placed in one of the four quadrants of the pool and underwent 10 trials per day in an interleaved fashion. Each trial lasted for 60 s and the rat was left to rest on the platform for 30 s. Latency time and path length reaching the platform were recorded and calculated using the Watermaze^TM^ software (Actimetrics Inc., IL, USA).

### 3.2 MRI

MRI was acquired on a 9.4 T system using a volume coil (Rapid Biomedical GmbH, Germany) for transmission and a custom-designed 1.5-cm surface coil for reception. Initially, the rats were anesthetized with 2–3% isoflurane in air and O_2_ (47% O_2_) mixture. After securing the head in a MRI compatible stereotaxic holder, 0.05 mg/kg medetomidine (Dormitor®, Pfizer, USA) was given intraperitoneally after which sedation was maintained with 0.1 mg/kg/h continuous infusion and isoflurane was switched off 15 min after. Respiration rate was monitored and rectal temperature was controlled at 37 °C using a MRI-compatible air heater (SA Instruments Inc., NY, USA). The rsMRI was acquired 40 min after the start of medetomidine infusion using single-shot spin-echo EPI with TR/TE=500/30 ms, thickness=1 mm, gap=0.1 mm, matrix=64×64, FOV=25.6×25.6 mm^2^ and 6 axial (i.e. coronal in rodent brain) slices centered at the hippocampus. This leads to an in-plane resolution of 0.4×0.4 mm^2^. A total of 1200 volumes were acquired in 10 min. High-resolution (0.1×0.1 mm^2^, thickness=1 mm, gap=0.1 mm) structural MRI was acquired over the whole brain using fast spin-echo with TR=2.5 s and TE=40 ms. The total time under sedative in MRI was <1.5 h.

**Fig. 1:**
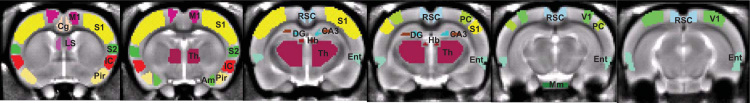
Location of ROI. The 34 ROIs chosen for calculating the correlation matrix and network plot.

**Fig. 2:**
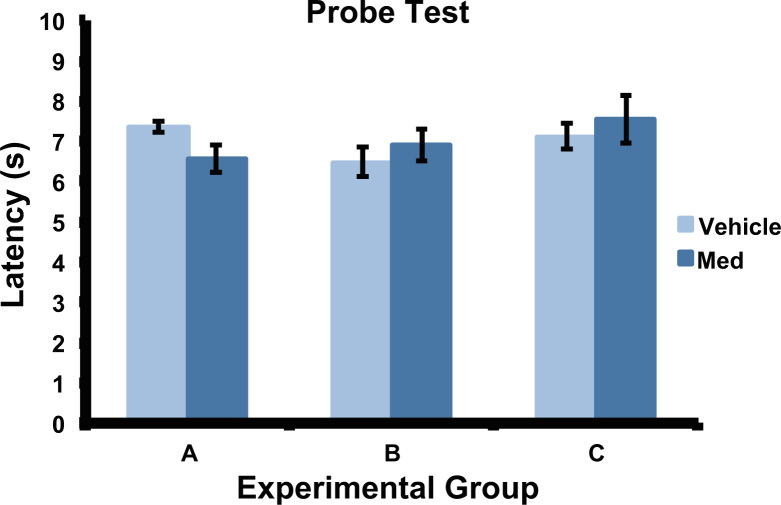
Probe tests of medetomidine effect. Latency time of the probe tests conducted in animal injected with medetomidine or saline infused on day 1 after 5-day of training (group A), on day 7 after training (group B), and on both day 1 and day 7 after the training (group B). No difference was seen in the latency times between the saline (7.36±0.13 s) and medetomidine (6.64±0.35 s; *p*=0.6) injected rats in group A; between saline (6.48±0.35 s) and medetomidine (6.9±0.4; *p*=0.4) injected rats in group B; and between saline (7.12±0.31 s) and medetomidine (7.54±0.58; *p*=0.8) injected rats in group C.

MRI was processed using FSL (http://fsl.fmrib.ox.ac.uk/fsl) and Matlab (Mathworks, MA, USA). Data was then filtered at 0.01–0.1 Hz. Signal from the ventricle was regressed to suppress physiological artifacts. An ROI of 2×2 pixels was manually selected from a ventricle of large enough size in each slice. The averaged EPI of each animal was first registered to its own structural MRI and then a stereotaxic MRI template of 0.2 mm isotropic resolution [2] using linear and nonlinear registration. Then spatial Gaussian smoothing (FWHM=0.4 mm) was applied. Seed-based correlation analysis was used to detect functional connectivity. Thirty-two a piori regions-of-interest (ROIs) of 2×2 pixels were defined on the stereotaxic template based on anatomical structures comprising the spatial memory networks [3], including the left and right lobes of primary somatosensory (S1), secondary somatosensory (S2), cingulate (Cg), retrosplenial (RSC), parietal (PC), entorhinal (Ent), piriform (Pir), insular (IC), motor (M1), visual (V1) cortices, as well as, amygdala (Am), thalamus (Th), mammillary nuclei (Mm), habenula (Hb), lateral septal nucleus (LS), dentate gyrus (DG), and hippocampal CA3 (CA3) (Fig. 1). The reference time courses from the 34 ROIs were then correlated with the time courses throughout the brain to generate the functional correlation maps (Fig. 2).

**Fig. 3:**
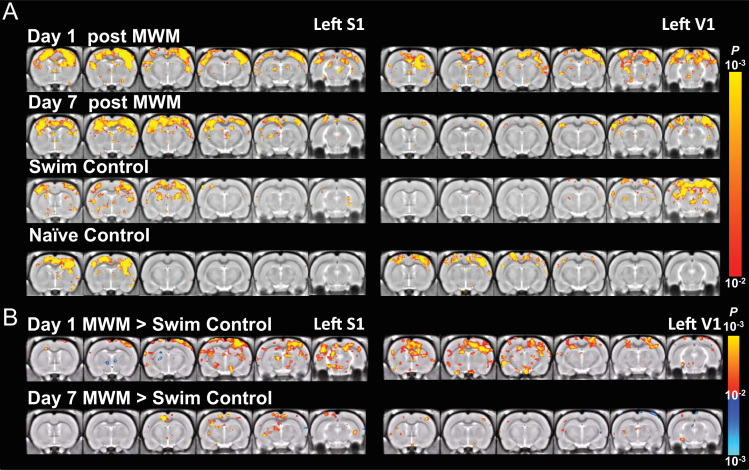
S1 and V1 functional correlation maps of 5-Day MWM trained and control rats. (A) The correlation with respect to left S1 (left column) and left V1 (right column) in rats (from top to bottom) on day 1 after 5-day training in MWM, day 7 after training, day 1 after swim control, and home cage (naïve) control (*p*<0.01, one-sample *t*-test, corrected by FDR). (B) The difference of connectivity between the MWM trained and the swim control groups on day 1 and day 7 after the 5-day training (*p*<0.01, 2-sample *t*-test, corrected by FDR).

**Fig. 4:**
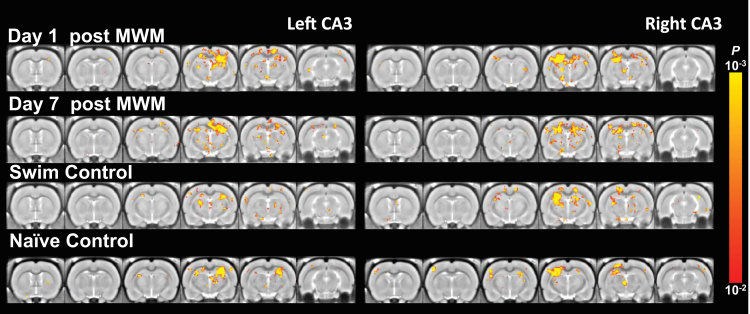
CA3 functional correlation maps of 1-Day MWM trained and control rats. The connectivity with respect to left CA3 (left column) and right CA3 (right column) in rats (from top to bottom) on day 1 after 1-day training in MWM, day 7 after training, day 1 after swim control, and home cage (naïve) control (*p*<0.01, one-sample *t*-test, corrected by FDR).

**Fig. 5:**
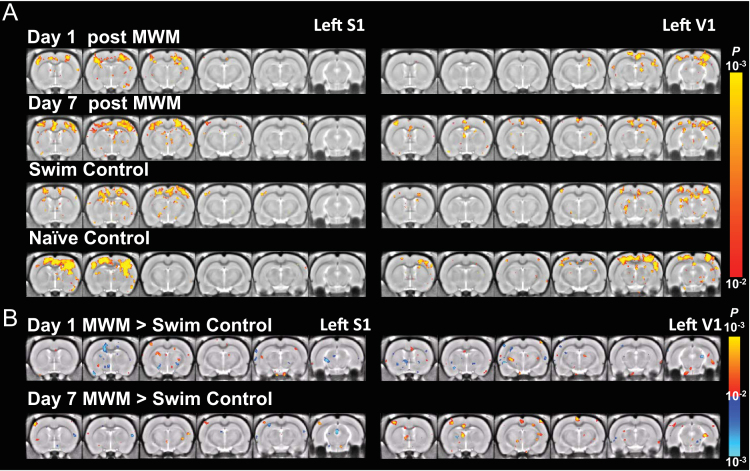
S1 and V1 functional correlation maps of 1-Day MWM trained and control rats. (A) The correlation with respect to left S1 (left column) and left V1 (right column) in rats (from top to bottom) on day 1 after 1-day training in MWM, day 7 after training, day 1 after swim control, and home cage (naïve) control (*p*<0.01, one-sample *t*-test, corrected by FDR). (B) The difference of connectivity between the MWM trained and the swim control groups on day 1 and day 7 after the 1-day training (*p*<0.01, 2-sample *t*-test, corrected by FDR).

**Fig. 6:**
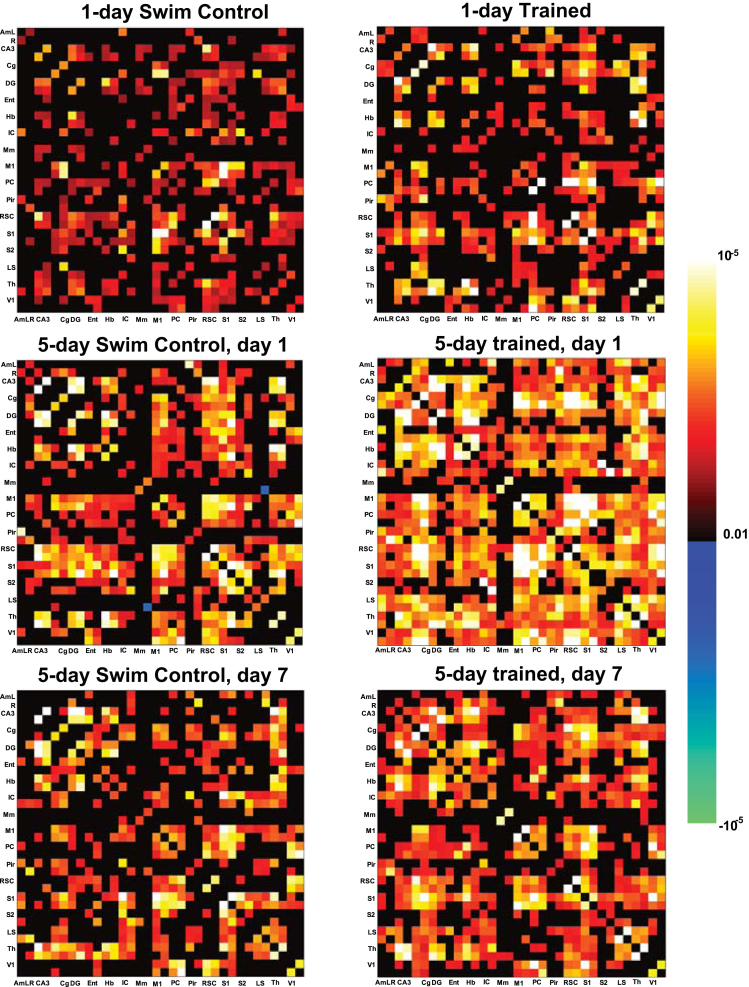
Correlation matrices of swim control and MWM trained rats. The correlation among the 34 ROIs from the swim control (left column) and MWM trained rats (right column) with (from top to bottom) 1-day of training, 5-day of training, and 7 days after 5-day of training (*p*<0.01, one-sample *t*-test, uncorrected).

**Fig. 7:**
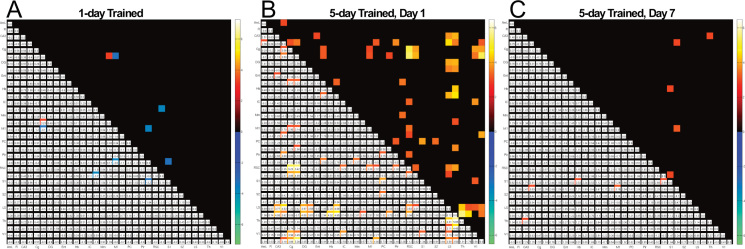
Connectivity matrix in 1-day and 5-day trained rats. The correlation matrix among 36 regions in the brain shows (A) low connectivity at day-1, (B) extensive connectivity 1 day after 5 day training, and (C) reduced connectivity after 7 days in the 5-day trained rats compared to the swim control (*p*<0.05, two-sample *t*-test, uncorrected).

**Fig. 8:**
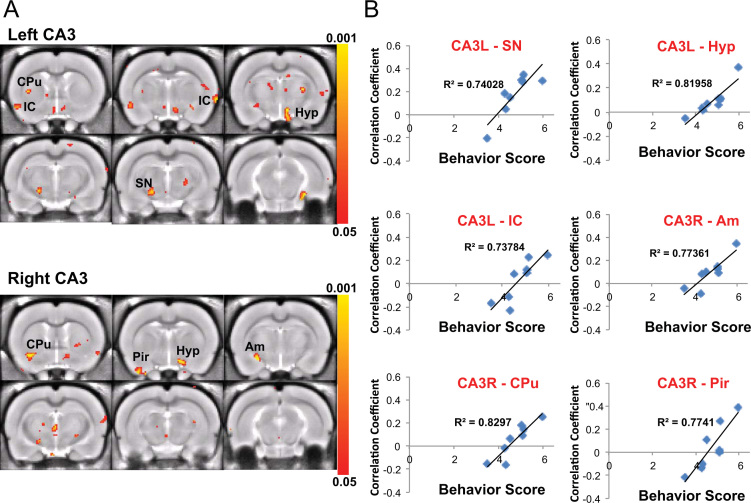
Behavioral correlate with CA3 connectivity in 1-day trained rats. (A) CA3 correlation maps show regions with significant correlation with the behavioral score in 1-day trained rats (*p*<0.05, FDR corrected). (B) The connectivity strength (correlation coefficient) between CA3 and SN, hypothalamus (Hyp), IC, Am, CPu, Pir are highly correlated with the learning performance.

**Fig. 9:**
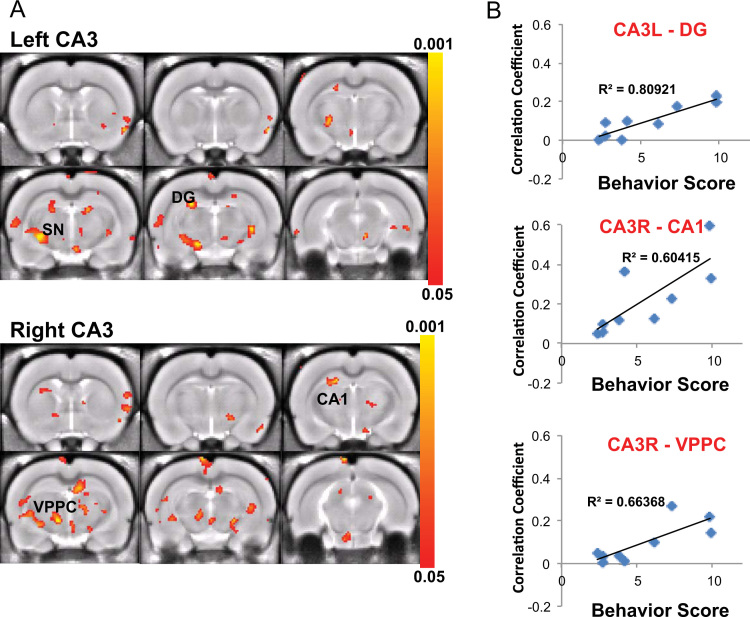
Behavioral correlate with CA3 connectivity in 5-day trained rats. (A) CA3 correlation maps show regions with significant correlation with the behavioral score in 5-day trained rats (p<0.05, FDR corrected). (B) The connectivity strength (correlation coefficient) between CA3 and SN, hypothalamus (Hyp), IC, Am, CPu, Pir are highly correlated with the learning performance.

**Fig. 10:**
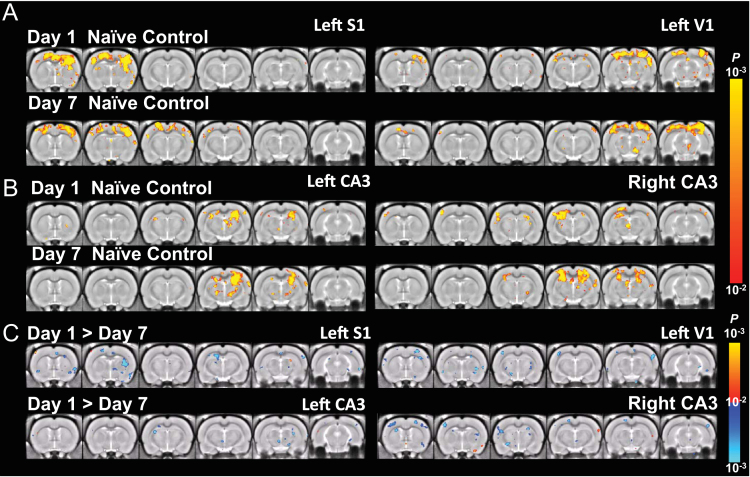
S1, V1, and CA3 functional correlation maps of Home Cage Control rats. The connectivity with respect to (A) left S1 (left column) and left V1 (right column) and (B) left CA3 (left column) and right CA3 (right column) in rats from the home cage (naïve) control group scanned on day 1 and day 7 (*p*<0.01, one-sample t-test, corrected by FDR). (C) The difference of connectivity between the day 1 and day 7 (*p*<0.01, 2-sample *t*-test, corrected by FDR).

For group statistics, correlation was transformed by Fisher z-transform. Group functional correlation maps was calculated by one-sample *t*-test on the z maps in the group. Second-level analysis was conducted by two-sample *t*-test between groups. All maps were thresholded at *p*<0.01, corrected by False Discovery Rate for multiple comparison [4]. (Fig. 3, Fig. 4, Fig. 5, Fig. 6, Fig. 7, Fig. 8, Fig. 9, Fig. 10).

## References

[1] Vorhees C., Williams M. (2006). Morris water maze: procedures for assessing spatial and related forms of learning and memory. Nat. Protoc..

[2] Schweinhardt P., Fransson P., Olson L., Spenger C., Andersson J. (2003). A template for spatial normalisation of MR images of the rat brain. J. Neurosci. Methods.

[3] Conejo N. (2010). Spatial learning of the water maze: Progression of brain circuits mapped with cytochrome oxidase histochemistry. Neurobiol. Learn. Mem..

[4] Genovese C., Lazar N., Nichols T. (2002). Thresholding of statistical maps in functional neuroimaging using the false discovery rate. Neuroimage.

