# Supplementary material for: Resting state functional connectivity data supports detection of cognition in the rodent brain
Source: Data Brief. 2016 Mar 15;7:1156–64. doi: 10.1016/j.dib.2016.03.041 (PMC4833131; doi:10.1016/j.dib.2016.03.041)
Supplement: Supplementary file 1 — Supplementary material [file mmc1.doc]

7^th^ March 2016

Dear Section Editor:

The authors have no conflict of interest to declare

Yours sincerely,

Fatima A Nasrallah, Ph.D.

Head, Queensland Brain Institute,

University of Queensland

Australia

Adjunct Professor, Clinical Imaging Research Center,

NUS/A*STAR, Singapore
